# Proteomic Characterisation of Heart Failure Reveals a Unique Molecular Phenotype for Hypertrophic Cardiomyopathy

**DOI:** 10.3390/biomedicines12081712

**Published:** 2024-08-01

**Authors:** Claire Tonry, Katie Linden, Patrick Collier, Mark Ledwidge, Ken McDonald, Ben C. Collins, Chris J. Watson

**Affiliations:** 1 Wellcome-Wolfson Institute for Experimental Medicine, Queen’s University Belfast, Belfast BT9 7BL, UK; claire.tonry@qub.ac.uk (C.T.);; 2 Department of Cardiovascular Medicine, Cleveland Clinic, OH 44195, USA; 3 STOP-HF Unit, Department of Cardiology, St. Vincent’s University Hospital Healthcare Group, D04 T6F4 Dublin, Ireland; 4 UCD Conway Institute of Biomolecular and Biomedical Research, School of Medicine, University College Dublin, D04 V1W8 Dublin, Ireland; 5 School of Biological Sciences, Queen’s University Belfast, Belfast BT9 5DL, UK

**Keywords:** hypertrophic cardiomyopathy, biomarkers, mass spectrometry, proteomics

## Abstract

Hypertrophic cardiomyopathy (HCM) is a disease, which is difficult to diagnose at an early stage and for which there is a pressing need for more effective treatment options. The purpose of this study was to compare the molecular profile of HCM to that of ischaemic cardiomyopathy (ISCM) and dilated cardiomyopathy (DCM) for identification of protein and pathway targets that could support the development of better diagnostic and treatment options for HCM. A high-throughput mass spectrometry workflow was applied to achieve deep quantitative coverage of left ventricular tissue from HCM, DCM, ISCM and non-heart-failure control patients. HCM had a diverse proteomic profile compared to that of DCM and ISCM. Differentially expressed proteins unique to HCM were identified based on an observed fold change of ≥1.5 or ≤0.67 and q-value ≤ 0.05. Candidate proteins of interest were found to be significantly associated with clinical features of HCM. The significant association between these proteins and HCM was validated in an independent dataset. This represents one of the largest and deepest proteomic datasets for myocardial tissue reported to date. The dataset highlights the diverse proteomic profile of HCM, relative to other cardiomyopathies, and reveals disease-relevant pathways and promising biomarker candidates that are uniquely associated with HCM.

## 1. Introduction

Heart failure is a complex clinical syndrome characterised by symptoms and signs including breathlessness, peripheral oedema, pulmonary oedema and fatigue. It can be caused by a range of different structural and functional cardiac abnormalities that share the common endpoints of elevated intracardiac pressures and inadequate cardiac output [1]. Heart failure is a massive population health issue, with more than 10% of individuals over the age of 75 being affected, which will only increase with our current ageing population [2]. The current cost of treating heart failure is estimated to be around 3% of the total health spend, and this cost is predicted to increase by at least 200% in the next 20 years [3].

There are many varied underlying pathological processes that can result in the structural and functional changes that cause heart failure, and these include ischaemic heart disease, valvular heart disease, diabetes, obesity, inherited cardiomyopathies and toxic injury from drugs such as chemotherapy agents [4]. Ischaemic cardiomyopathy (ISCM), where heart failure develops as a result of coronary heart disease with myocardial injury as a result, is the most common aetiology of heart failure worldwide [5]. Dilated cardiomyopathy (DCM) describes a heterogenous group where heart failure is the final common outcome of a range of non-ischaemic insults including genetic and environmental factors [6]. Hypertrophic cardiomyopathy (HCM) is the most common inherited cardiac condition that affects our population and is characterised by cardiac hypertrophy, which can result in left ventricular outflow tract obstruction in more severe cases [7]. Although HCM is recognised to be a genetically inherited condition, only around 50% of patients diagnosed with this condition are identified as carrying a single pathogenic sarcomeric gene mutation [8]. While gene-positive patients are known to develop a more significant phenotype of the disease with higher rates of heart failure and sudden cardiac death (SCD), little is known about other factors that control this variable phenotypic expression in HCM [9]. Proteomic characterisation may offer further insights into the underlying mechanism of this variability. 

At present, heart failure diagnosis and monitoring include biomarkers such as natriuretic peptides that may give an indication of whether there is pressure or volume overload of the heart but are non-specific and so do not offer any insight into the aetiology of the underlying pathological process causing the heart failure [10]. This study set out to characterise the differences in cardiac tissue protein signatures across varying heart failure aetiologies to identify unique protein and pathway targets that could support HCM-specific biomarker development. 

## 2. Materials and Methods

### 2.1. Sample Cohort

Left ventricular (LV) septal tissues were collected from 39 male patients who underwent orthotropic cardiac transplantation for ISCM (n = 9), DCM (n = 9) or septal myectomy for symptomatic obstructive hypertrophic cardiomyopathy (oHCM; n = 12), and in matched control patients with non-failing hearts (n = 9) who died of noncardiac causes. The study conformed to the principles outlined in the Declaration of Helsinki, and patients gave informed consent. Ethical approval for data collection and the use of tissue was obtained from the Cleveland Clinic Institutional Review Board. Patient demographics and clinical characteristics have previously been described [11].

### 2.2. Sample Preparation

Tissue samples were lysed in RIPA buffer. Protein samples were quantified using the bicinchoninic acid (BCA) assay, as per the manufacturer’s instructions. A mass of 100 μg of protein was digested with a 1:50 protein–enzyme ratio of LysC (FUJIFILM Wako) Laboratory Chemicals, Richmond, VA, USA) and a 1:100 protein–enzyme ratio of Trypsin (Promega, Madison, WI, USA) as per the Filter-Aided Sample Preparation (FASP) method described previously [12]. Peptides were dried down under a vacuum, re-suspended in 1% trifluoracetic acid (TFA) and then de-salted on C18-packed stage-tip columns [13]. Briefly, C18 stage-tips were activated with 50 μL of 50% acetonitrile (AcN)/0.1% TFA. The stage-tips were washed with 1% TFA before adding 8 ug of peptide in 1% TFA. After a further two wash steps with 1% TFA, peptide was eluted from the stage-tip in 25 μL of 50% AcN/0.1% TFA. All digested samples from each disease and non-heart-failure group were pooled and fractionated using the Pierce ™ High-pH Reversed-Phase Peptide Fractionation Kit, as per the manufacturer’s instructions. Prior to mass spectrometry analysis, peptide samples were dried down under a vacuum and re-constituted in 2% AcN, 1% formic acid. Peptide samples were analysed on a nanodrop at A209 to ensure all samples were approximately 200 ng/μL.

### 2.3. Mass Spectrometry Analysis

Mass spectrometry (MS) proteomics data were acquired on a timsTOF Pro (Bruker, Billerica, MA, USA) quadrupole time-of-flight mass spectrometer integrating trapped ion mobility separations coupled online via a Captivespray electrospray source (Bruker) to a nanoElute (Bruker) nanoflow liquid chromatography system. Solvent composition at the two channels was 0.1% formic acid in water for channel A and 0.1% formic acid in ace-tonitrile for channel B. Peptides were separated on a 25 cm × 75 µm Aurora packed emitter column (IonOpticks, Fitzroy, Australia) using the following gradient: 2% B, 0 min; 17% B, 60 min; 25% B, 90 min; 37% B, 100 min. The flow rate was 400 nL/min, and the column was maintained at 50 °C. MS data were acquired in data-independent acquisition mode using the ‘standard diaPASEF’ windows scheme (25 *m*/*z* width, 4 windows per 100 ms ion mobility scan, 16 ion mobility scans per duty cycle) as described in Meier et al. [14] and in data-dependent acquisition mode as described in Meier et al. [15] (100 ms ion mobility scan, 10 ion mobility scans per cycle). Analysis of high-pH reversed-phase fractionated sample pools from each sample group (n = 32 fractionated samples) was performed in ddaPASEF mode to generate spectral library data. A technical replicate (TR) of the samples, generated from pooling all samples post-stage-tipping, was analysed at the beginning, middle and end of the run to monitor technical reproducibility. A sample pool (SP), generated from pooling all samples pre-digest, was analysed at regular intervals throughout the run in diaPASEF mode to monitor reproducibility of the sample preparation process.

### 2.4. Data Analysis

Spectronaut 18 software was used for spectral library building and protein identifications. The .dda files generated from analysis of TR samples and fractionated samples were used for spectral library building. The Homo Sapien proteome (UP000005640) was used as a reference proteome for spectral library building. The diaPASEF files were processed against the resulting spectral library for protein identification with an FDR cutoff set to ≤0.05. Significant protein candidates were identified based on a fold change of ≥1.5 or ≤−1.5 and q-value ≤ 0.005. The resulting dataframe of protein identifications was further processed in R (4.3.2) to filter out candidates with >30% missing values in the matrix and impute the remaining missing values based on mean expression. All data were log transformed prior to further analysis. Independent Student’s *t*-test and ANOVA with FDR correction (rstatistix) were used to further refine the selection of candidates based on complete expression data. Candidates were triaged based on corresponding CV values calculated from SP samples. Pathway analysis of candidates of interest was performed using the pathfinder package. SPSS v29 software was used to identify protein candidates with best performance for prediction of oHCM. Prism v10 software was used for correlation analysis. For all analyses, non-parametric tests were applied if data were found to be non-normally distributed. 

## 3. Results

### 3.1. Proteomic Characterisation of Heart Failure

**Figure 1:**
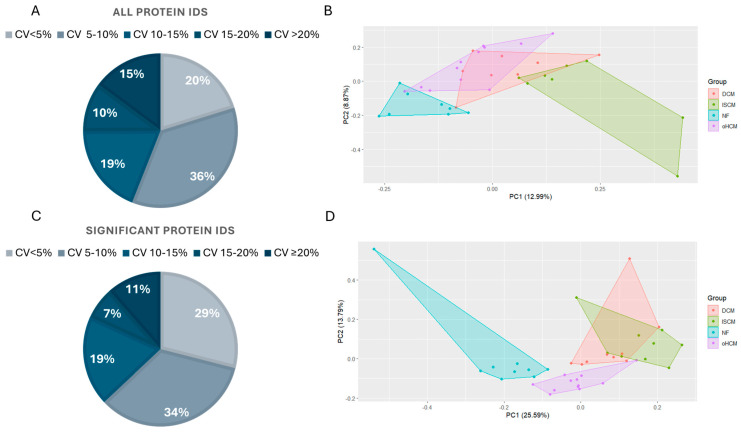
Proteomic characterisation of heart failure aetiologies. The majority of proteins in the complete (>70% complete) dataset (n = 7146 proteins) were identified in sample pool replicates with a %CV < 20% (**A**). Principal component analysis demonstrates the overlap in protein expression profiles between non-failing heart (NF) and heart failure (HF) aetiologies (**B**). ANOVA analysis with Tukey post hoc analysis applied for multiple group comparisons highlighted proteins that are significantly differentially expressed between all groups. The majority of these proteins were measured in sample pool replicates with a %CV < 15% (**C**). ANOVA analysis of proteomic changes between HF aetiologies only reveals a unique proteomic profile for oHCM (**D**). DCM = dilated cardiomyopathy; ISCM = ischaemic cardiomyopathy; NF = no heart failure; oHCM = obstructive hypertrophic cardiomyopathy.

Samples from 30 patients affected with heart failure with varying aetiologies including oHCM, DCM and ISCM were analysed along with age- and sex-matched non-heart-failure controls with demographics as described in a previous publication by Glezeva et al. [11]. Label-free mass spectrometry analysis resulted in the identification of 7683 proteins across all samples, with 7146 of those being detected in >70% of samples. Data from the sample pool samples analysed throughout the run were used to calculate the coefficient of variance (CV) in the refined (>70% complete) protein dataset, before missing values were imputed. The majority (>85%) of proteins were measured with a CV of less than 20%, and the greatest proportion (36%) of proteins were measured with a CV less than 10% (Figure 1A). Principal component analyses of the full dataset separated non-heart-failure patients’ proteome from the three heart failure disease proteomes, although there was substantial overlap in protein expression between the three HF subtypes (Figure 1B). ANOVA analysis with Tukey HSD post hoc correction revealed 1497 significantly differentially expressed proteins between the four groups. Over 80% of these proteins were measured with a CV less than 15% (Figure 1C). The PCA analysis based on this subset of significantly differentially expressed proteins revealed a unique proteomic signature for oHCM, relative to the other HF subtypes (Figure 1D).

### 3.2. Differential Protein Expression between Heart Failure and Non-Failing Heart Tissue

**Figure 2:**
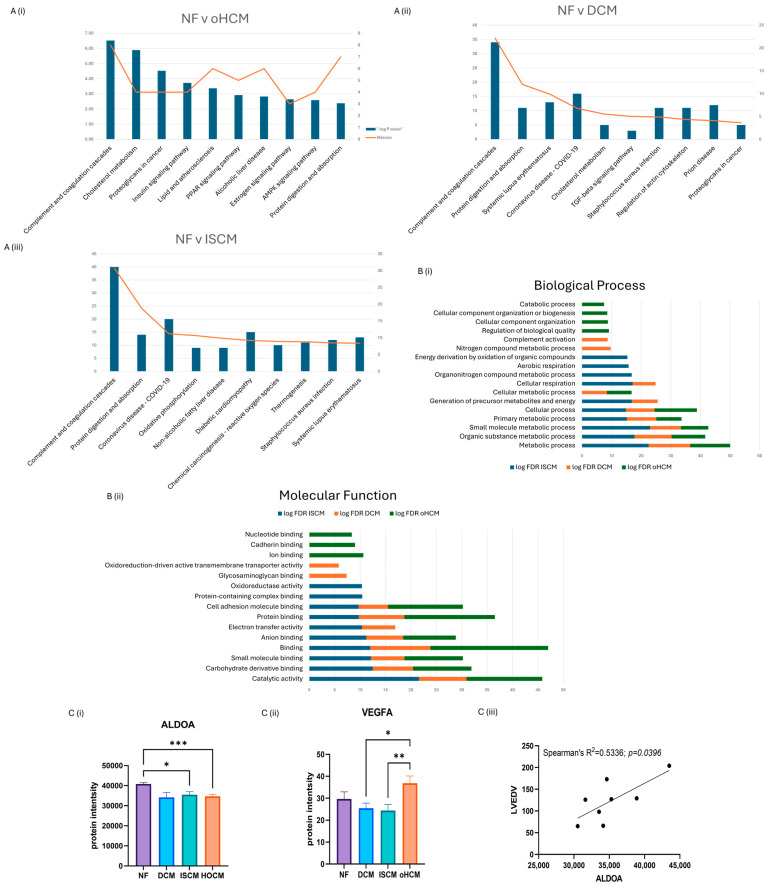
Significant protein expression changes associated with heart failure. Independent *t*-test analysis was applied to identified significant protein changes between each heart failure (HF) aetiology and control non-failing hearts (NF) (*p* ≤ 0.05). The top ten KEGG pathways enriched by differential protein expression in each aetiology were identified (**A**). A number of biological processes and molecular functions were identified that were unique to oHCM based on STRING analysis of significant proteins (unadjusted *p* ≤ 0.05) (**B**). Involvement of AMPK and angiogenesis pathways in oHCM is reflected with de-regulated expression of ALDOA and VEGFA (**C**(**i**,**ii**)). ALDOA expression is positively correlated with left ventricular end diastolic volume (LVEDV; **C**(**iii**)). ALDOA bar chart = mean ± SEM from Brown–Forsythe and Welch ANOVA test. Bar plot for VEGFA = mean ± SEM independent *t*-test with Welch’s correction applied: oHCM vs. each patient group. DCM = dilated cardiomyopathy; ISCM = ischaemic cardiomyopathy; NF = no heart failure; oHCM = obstructive hypertrophic cardiomyopathy. * = *p* < 0.05, ** = *p* < 0.01, *** = *p* < 0.0001.

Over 150 significantly differentially expressed proteins were identified between the different heart failure aetiologies and the non-heart-failure control (q-value ≤ 0.05). Differentially expressed proteins were identified in oHCM (n = 39), DCM (n = 6) and ISCM (n = 103) when compared to the non-heart-failure controls (*p* ≤ 0.05). The top canonical pathways associated with each disease subtype were recorded. DCM and ISCM shared 2 of their top 10 pathways, with the complement system being most strongly associated pathway in both aetiologies and ‘protein digestion and absorption’ being the second most enriched. The complement system was also enriched by oHCM-associated proteins; however, in contrast to DCM and ISCM, the second most enriched pathway was that of ‘cholesterol metabolism’ (Figure 2A). Molecular functions and biological processes most enriched in all three aetiologies were identified from STRING analysis of significant proteins (unadjusted *p* ≤ 0.05), revealing processes and functions that were differentially enriched for each aetiology (Figure 2B). Significant proteins were further refined with application of an FDR-adjusted *p*-value cut-off of ≤0.05. Thirty-two proteins were uniquely differentially expressed in oHCM with five proteins unique to DCM and ninety-four proteins unique to ISCM. It is notable that the majority of significant proteins identified in association with each aetiology were unique to that aetiology, i.e., 94/103 (91%) ISCM-associated proteins were found to be uniquely associated with ISCM, 5/8 (63%) DCM-associated proteins were found to be uniquely associated with DCM and 32/39 (82%) oHCM-associated proteins were found to be uniquely associated with oHCM.

### 3.3. Differential Protein Expression in Obstructive HCM Reveals Unique Pathology of Disease

**Figure 3:**
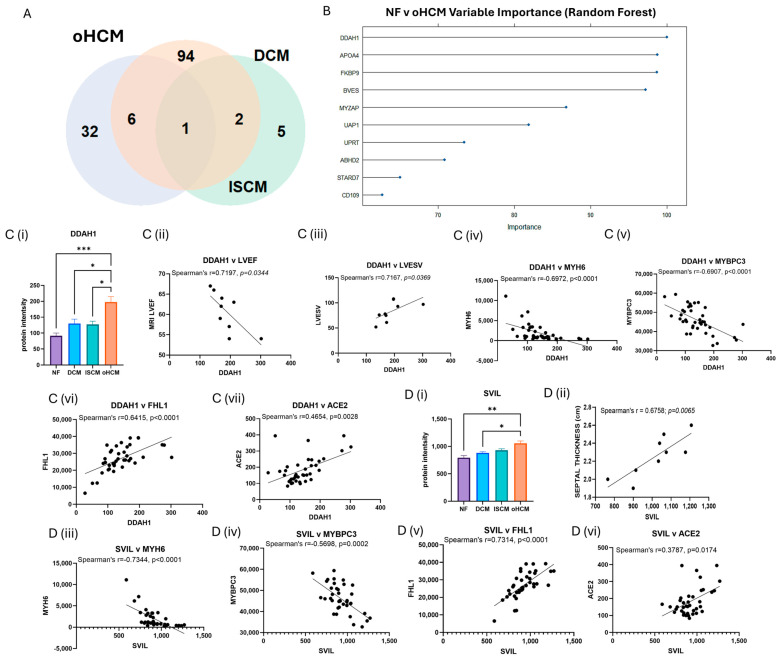
Disease-relevant differential protein expression changes in obstructive HCM. FDR correction was applied for more stringent selection of significant proteins identified from Figure 2. Thirty-two proteins were identified with significant differential expression (FDR-adjusted *p* ≤ 0.05) were associated with oHCM only (**A**). The top 10 differentially expressed proteins with the highest importance for differentiation between non-failing hearts (NF) and oHCM were identified from random forest analysis (**B**). The top protein, DDAH1 is significantly associated with left ventricular ejection fraction (LVEF) and left ventricular end systolic volume (LVESV) in oHCM patients. DDAH1 is also associated with Myosin 6, myosin-binding protein C3 (MYBPC3), four and a half LIM domains protein 1 (FHL1) and angiotensin-converting enzyme 2 (ACE2) (**C**(**i**)–**C**(**vii**)). SVIL was uniquely associated with oHCM and also significantly correlated with septal thickness. SVIL is also correlated with MYH6, MYBPC3, FHL1 and ACE2 (**D**(**i**)–**D**(**vi**)). Bar plots show mean ± SEM from Brown–Forsythe and Welch ANOVA test with Dunnett’s T3 multiple-comparisons test applied. * = *p* < 0.05, ** = *p* < 0.01, *** = *p* < 0.0001.

The full list of 32 proteins that are uniquely associated with oHCM is available in Supplementary Table S1. Several oHCM-associated proteins were found to correlate significantly with clinical features relevant to oHCM disease (Table 1). DDAH1 is one such protein that was found to be significantly up-regulated in oHCM and significantly negatively correlated with left ventricular ejection fraction (LVEF) and significantly positively correlated with left ventricular end systolic volume (LVESV, Figure 3C). ALDOA was significantly down-regulated in oHCM compared with non-heart-failure controls. ALDOA expression in oHCM patients was significantly associated with right ventricular systolic pressure (RVSP) and left ventricular end diastolic volume (LVEDV, Figure 2C(iii)). ALDOA is known to have a role in AMPK signalling and is also associated with VEGF signalling. There was no significant association between ALDOA protein expression and expression of AMPK in this dataset; however, the AMPK pathway was one of the top 10 most significantly enriched pathways in oHCM (Figure 2B). There was no significant correlation between expression of ALDOA or VEGF protein either; however, a non-significant increase in VEGF was observed in oHCM patients relative to non-heart-failure controls, whereas the opposite trend was observed for ISCM and DCM patients (Figure 2C). Another unique oHCM-associated protein of interest that was identified was SVIL, which was found to be significantly associated with septal thickness in oHCM patients (Figure 3D). A refined list of the top up- and down-regulated candidate proteins of interest that were identified from comparison of non-heart-failure controls and oHCM samples and their association with cardiac function is available in Supplementary Table S3. Significantly differentially expressed proteins unique to oHCM that were validated in an independent dataset are shown in Table 1.

### 3.4. Differential Protein Expression between Obstructive HCM and Other Heart Failure Subtypes

**Figure 4:**
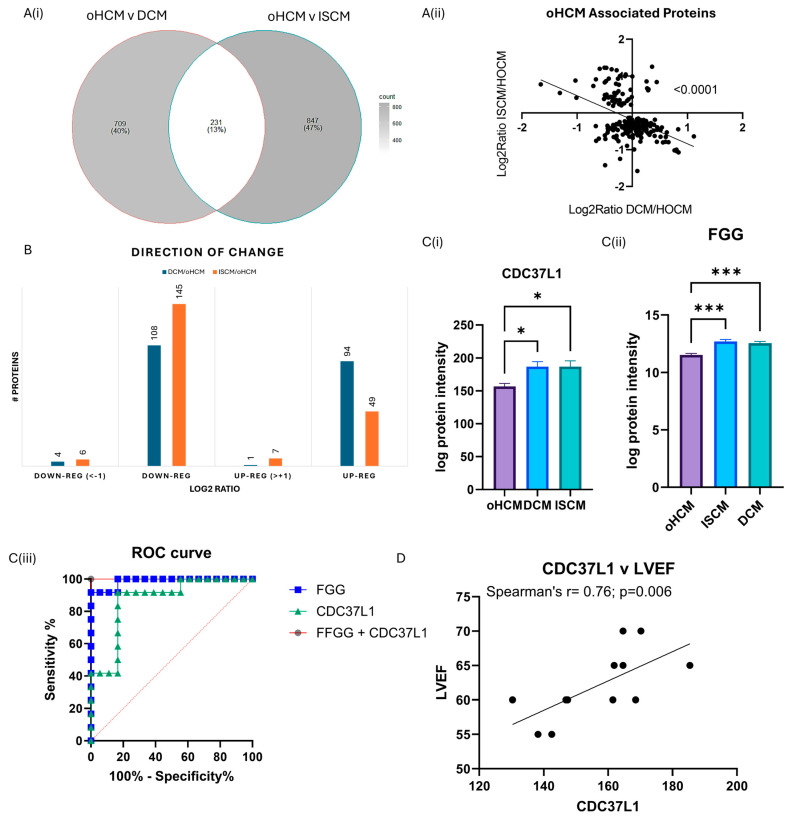
Diverse proteomic profile of obstructive HCM compared with DCM and ISCM. The oHCM proteome was compared to the proteome of patients with ISCM and DCM to identify differentially expressed proteins (independent *t*-tests; *p* ≤ 0.05) (**A**(**i**)). Proteins that are differentially expressed between oHCM and DCM and between oHCM and ISCM were consistent in the direction of change between oHCM and each other aetiology (**A**(**ii**)). The majority of differentially expressed proteins are down-regulated in oHCM (**B**). Logistic binary regression analysis with forward:wald selection identified two proteins, CDC37L1 (**C**(**i**)) and FGG (**C**(**ii**)), which are highly predictive of oHCM within a cohort of HF patients (**C**(**iii**)). CDC37L1 is strongly correlated with left ventricular ejection fraction, a parameter used in diagnosis of oHCM (*p* = 0.006) (**D**). LVEF = left ventricular ejection fraction. Bar plots show mean ± SEM from Brown–Forsythe and Welch ANOVA test with Dunnett’s T3 multiple-comparisons test applied. * = *p* < 0.05, *** = *p* < 0.0001.

One-way ANOVA analysis was performed to identify protein changes between oHCM, ISCM and DCM patients. The greatest number of protein changes were identified between oHCM and ISCM, and there were 204 proteins significantly differentially expressed between both DCM and ISCM and oHCM patients (Figure 4A, Supplementary Table S2). The direction of change was consistent in DCM and ISCM patients (Figure 4B). The majority of differentially expressed proteins were down-regulated in oHCM, compared to DCM and ISCM (Figure 4B). Of these ‘common oHCM-associated’ proteins, the combination of the proteins Fibrinogen gamma-chain (FGG) and Hsp90 co-chaperone Cdc37-like 1 (CDC37L1) was identified based on a binary logistic regression model with forward:wald selection as the optimal combination for differentiation between oHCM and either DCM or ISCM patients (Figure 4C). Repeating the forward:wald selection with either of these proteins removed from the list of input variables consistently identified an optimal predictive model that contained at least one of either FGG or CDC37L1. CDC37L1 was significantly positively correlated with LVEF in this cohort of oHCM patients (Figure 4D; *p* = 0.006). A number of the other oHCM-associated proteins also associate significantly with clinical features of the disease (Table 2).

### 3.5. In Silico Validation of HCM-Associated Proteins 

**Figure 5:**
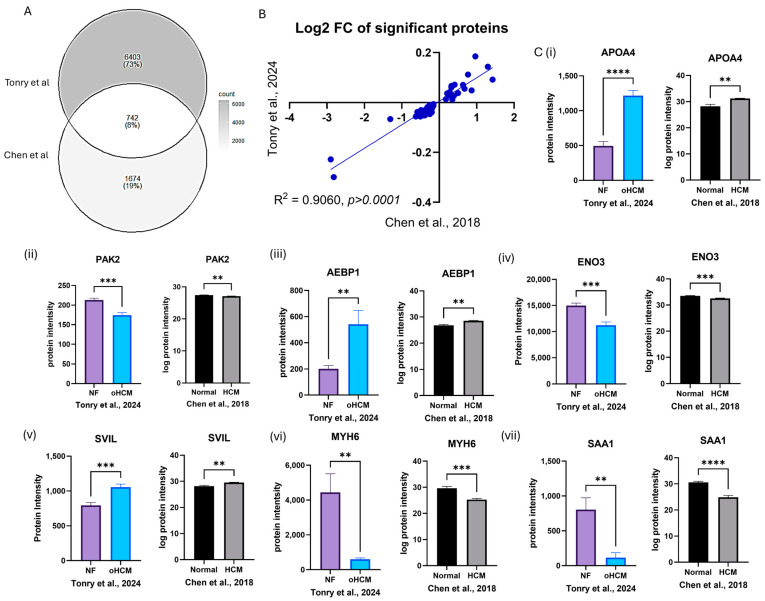
In silico validation of HCM-associated protein expression changes. Protein expression data from analysis of left ventricular tissue in an independent dataset were used to validate protein expression changes reported from this study (**A**). The ratio of expression of significantly differentially expressed proteins between HCM and NF patients was consistent in both datasets (**B**). Proteins that were uniquely differentially expressed between HCM and NF were validated in the Chen et al. [16] dataset (**C**(**i**)–(**vii**)). Bar plots show the mean ± SEM from results of independent *t*-tests with Welch’s correction. Black and white bars = Chen et al. dataset [16]; coloured bars = Tonry et al. dataset. ** = *p* < 0.05, *** = *p* < 0.01, **** = *p* < 0.0001.

A previously published mass spectrometry dataset was accessed from PRIDE (PXD008934) [16], which contained protein expression data from patients with hypertrophic cardiomyopathy (HCM; n = 9), ischaemic cardiomyopathy (ISCM; n = 6) and dilated cardiomyopathy (DCM; n = 5) in addition to non-heart-failure (normal; n = 7) controls. There was only 8% overlap in proteins identified between the dataset published by Chen et al. [16] and proteins identified in this study, due to the increased coverage of the ventricular tissue proteome that has been achieved here (Figure 5A). However, the data available were sufficient to validate some proteins that were found to be uniquely differentially expressed between HCM and non-heart-failure controls (Figure 5C, Table 1) and between HCM and ISCM/DCM (Supplementary Table S2). 

## 4. Discussion

This study represents one of the most in-depth proteomic characterisations of human cardiac tissue and reveals unique molecular features associated with obstructive hypertrophic cardiomyopathy. This insight is important as, while HCM is considered a genetically driven disease, the ability to predict clinical outcomes or offer personalised treatment approaches based on specific gene mutations remains limited for patients with HCM [17]. Several studies have been conducted with the aim of better understanding the underlying pathology of HCM, through proteomics-based analysis of human tissue and blood samples [18,19,20,21], and this study contributes further knowledge to this field.

At a high level, the data reveal differences in pathways found to be associated with oHCM, compared with pathways associated with DCM and ISCM. While all three cardiomyopathies are highly associated with de-regulation of complement and coagulation cascades, cholesterol metabolism was also very highly de-regulated in oHCM. De-regulated metabolism has been identified as a feature of HCM in previous proteomics-based studies of the disease [18,19,21]. Another metabolism-related pathway, the AMP-activated protein kinase (AMPK) signalling pathway, was also among the top 10 de-regulated pathways associated with oHCM and was not amongst the top 10 de-regulated pathways for DCM and ISCM. AMPK is a sensor of cellular energy and nutrient status [22]. Reduced activation of AMPK has been reported to be a causative factor of myocardial hypertrophy and arrhythmias, and targeting the AMPK pathway has thus been shown to have therapeutic benefits in patients with PRKAG2 syndrome, a metabolic condition that causes an HCM phenotype [23]. Fructose-bisphosphate aldolase A (ALDOA), a protein that was uniquely significantly associated with oHCM in this study, has been investigated as a therapeutic target for cardiac hypertrophy, whereby knockdown of ALDOA represses development of cardiac hypertrophy through activation of the AMPK signalling pathway [22]. Contrastingly, in this cohort, expression of proteins associated with AMPK-encoding genes (PRKAA1, PRKAG1, PRKAA2, PRKAB1, PRKAB2 and PRKAG2) are significantly higher in oHCM patients, compared with other heart failure aetiologies, and expression of ALDOA is significantly down-regulated. Although this is in contrast with findings from previous studies where overexpression of ALDOA has been associated with cardiac hypertrophy and fibrosis, there was a significant positive correlation between ALDOA and left ventricular end diastolic volume (LVEDV, Figure 3), suggesting a clinically relevant role in the disease. ALDOA is also associated with vascular endothelial growth factor-A (VEGF-A) activity. While no correlation between ALDOA and VEGF-A was observed in this cohort, it was noted that VEGF-A was significantly up-regulated in oHCM, compared with DCM and ISCM, suggesting that de-regulation of the angiogenesis pathway may also be a key point of difference between oHCM and other heart failure aetiologies. 

Proteins were identified that are significantly associated with only with oHCM and not the other heart failure aetiologies. N(G),N(G)-dimethylarginine dimethylaminohydrolase 1 (DDAH1) is the critical enzyme for degradation of asymmetric dimethylarginine, which is a nitric oxide synthase (NOS) inhibitor [24]. Published data on proteomics-based characterisation of different regions of the heart reveal that DDAH1 is most highly expressed in the left ventricle [25]. Preclinical studies provide evidence that overexpression of DDAH1 protects from angiotensin-II-induced cardiovascular damage and attenuates left ventricular remodelling after acute myocardial infarction [26,27]. It has been suggested that the protective function of DDAH1 in cardiomyocytes is as a consequence of the associated increase in levels of NOS. In agreement with this, a non-significant positive correlation between DDAH1 and NOS was observed in oHCM patients. However, elevated DDAH1 was significantly associated with decreased LVEF and increased LVESV (Figure 3) in oHCM patients. It is possible that the increase in DDAH1 is occurring in response to decreased LVEF, and this reflects the protective effect of the protein that has been observed in preclinical studies; however, this will need to be explored further. Indeed, DDAH1 expression was significantly positively correlated with levels of angiotensin-converting enzyme 2 (ACE2), an enzyme with known cardioprotective effects. DDAHA1 is also significantly positively correlated with four and a half LIM domains protein 1 (FHL1), which has a role in sarcomere organisation, and significantly negatively correlated with Myosin-6 (MYH6) and myosin-binding protein C (MYBPC3; Figure 3). These observations suggest relevance of DDAH1 in the pathogenesis of oHCM, as opposed to the other two aetiologies studied (DCM and ISCM), for which there was no significant change observed in DDAH1 expression. Another protein that was uniquely associated with oHCM was supervilin (SVIL), which is increased in oHCM. SVIL is an actin-binding protein and here was found to be significantly positively correlated with septal thickness. This is an interesting finding as left ventricular hypertrophy with increased ventricular wall thickness is a key pathological feature of the disease process in HCM. Like DDAH1, it is also significantly correlated with MYH6 and MYBPC3 and significantly positively correlated with FHL1 and ACE2, suggesting that this protein is relevant to oHCM. Indeed, from a recent study by Tardros et al., who undertook the largest genome-wide association study of HCM reported to date using samples from the UK biobank, SVIL was identified as a novel HCM disease gene [28]. This protein and its role in oHCM and other types of HCM is worthy of future investigation. 

Proteins that were significantly differentially expressed between oHCM and either DCM or ISCM were also investigated. The direction of change for said proteins was common between oHCM and DCM and ISCM, with the majority of significantly differentially expressed proteins being down-regulated in oHCM. Expression levels of the proteins Fibrinogen gamma chain isoform gamma-A (FGG) and Hsp90 co-chaperone Cdc37-like 1 (CDC37L1) were found to be most powerful for differentiating oHCM from DCM and ISCM patients. Both proteins are down-regulated in oHCM (Figure 4). FGG forms part of the fibrin matrix and cleavage of FGG produces fibrin, the most abundant component of blood clots. In a previous proteomic study, assessing molecular changes in the pre- and post-operative period in tetralogy of Fallot and ventricular septal defect, it was observed that elevated pre-operative levels of FGG returned to normal levels post-operatively, thereby indicating a direct link between this protein and structural damage to the heart [29]. In this dataset, FGG is significantly positively correlated with MMP2 and MMP9. This is also reflective of the previously reported associations between FGG and epithelial to mesenchymal transition [30]. CDC37L1 is a co-chaperone protein with an implicated role in tau regulation [31]. It has also been found to have association with hepatitis B virus-associated hepatocellular carcinoma [32]. Transcriptomics analysis revealed that CDC37L1 may be a targetable transcription factor in heart failure caused by idiopathic dilated cardiomyopathy; however, in this cohort there was no significant difference in CDC37L1 expression between DCM and non-heart-failure patients. Here, we report that CDC37L1 is significantly down-regulated in oHCM, compared with other heart failure aetiologies. It is significantly positively correlated with LVEF and, based on data produced previously by Linscheid et al., is most highly expressed in the left ventricle of non-diseased hearts, suggesting that this protein has a pathophysiological role in this disease [25]. This may be of interest as, unlike other heart failure aetiologies, reduced ejection fraction is a less common finding in the setting of HCM, diastolic dysfunction being a more common clinical feature.

The association between a number of proteins identified here and HCM was confirmed from analysis of proteomic data published by Chen et al. [16]. This was an ideal dataset to use for in silico validation as similar patient cohorts were analysed, and such tissue samples are difficult to obtain. However, given the improved sensitivity of the mass spectrometry methods used here, there was not very much overlap in proteins identified in both studies—73% of proteins identified in our study were not identified by Chen et al. As expected, MYH6 was one protein that was found to be significantly down-regulated in oHCM in both datasets. A number of other novel proteins of interest, including SVIL, were also validated in the Chen dataset (Figure 5), and this is notable given the different sample cohorts, lab environment and analysis techniques used in these two independent studies. This in silico validation justifies further exploration of the potential role of these proteins in obstructive HCM and non-obstructive HCM.

Proteomic characterisation of cardiomyopathy to the extent demonstrated here is challenging due to the high dynamic range of the cardiac muscle proteome, which is largely dominated by high abundance of a small number of contractile proteins, and difficulties in obtaining enough relevant human tissue samples [33]. This study benefitted from application of data-independent acquisition (dia) coupled with a modern mass-selection method called parallel accumulation serial fragmentation (PASEF) [14]. The excellent sensitivity and reproducibility of this method is evidenced in the high number of protein identifications (>7000) and low CV values for identified proteins—only 15% of proteins were measured with CV > 20% (Figure 1). The quality of this mass spectrometry dataset gives credibility to the findings reported here, and this dataset will be a valuable resource for further in-depth molecular investigations of three different heart failure aetiologies. Given the scarcity of heart failure biopsy samples and the difficulty in obtaining left ventricular tissue samples from relevant non-heart-failure controls, it is a strength that our cohort included a good representation of each heart failure aetiology as well as non-failure controls and that a suitable independent dataset was identified to verify findings in similar patient groups. The study is limited by the fact that all patients included in the sample cohort were male. Hence, it was not possible to determine the effect of sex on protein expression levels. It is also a limitation that mutational status for oHCM patients was not available, and it was not possible to make inferences on the effect of known HCM gene mutations on protein expression. However, given the association between proteins of interest with clinical measures of cardiac function and with expression of proteins that are encoded by HCM-relevant genes, there is enough evidence here to confirm the potential clinical relevance of the reported findings.

## 5. Conclusions

In conclusion, this study highlights the diverse molecular profile of oHCM, compared with other underlying heart failure aetiologies. Pathways related to cellular metabolism appear to be especially relevant to oHCM and could be explored further as therapeutic targets for management of the disease. Proteins have been identified and verified as potential oHCM-specific candidate biomarkers for earlier diagnosis and prognosis of the disease. Further investigations of the relevance of these proteins with different subtypes of HCM will be of value, in addition to fully elucidating the mechanistic role of these proteins in the pathology of the disease. 

## Figures and Tables

**Table 1:** Selection of significantly up- and down-regulated proteins in obstructive HCM compared to NF controls.

	Protein Name (Gene ID)	Median (NF, DCM, ISCM, oHCM)	*p*	p.adj	log2ratio	Clinical Correlations (r)
Unique oHCM + validated	Apolipoprotein A-IV (APOA4) *	1067.9	6.00 × 10^−7^	0.004	1.299	
	N(G),N(G)-dimethylarginine dimethylaminohydrolase 1 (DDAH1) *	132.4	4.00 × 10^−5^	0.027	1.116	LVEDV: r = −0.9048, *p* = 0.0046
						LVEF: r = −0.7197, *p* = 0.0344
						LVESV: r = 0.7167, *p* = 0.0369
	Fructose-bisphosphate aldolase A (ALDOA)	36,406.44	1.00 × 10^−4^	0.045	−0.235	LVEDV: r = 0.8333, *p* = 0.0154
	Ubiquitin domain-containing protein UBFD1 (UBFD1)	182.3	1.00 × 10^−4^	0.045	−0.487	
	Serine/threonine-protein kinase PAK 2 (PAK2)	197.35	2.00 × 10^−4^	0.047	−0.289	
	Beta-enolase (ENO3)	12,210.88	2.00 × 10^−4^	0.047	−0.419	
	Large ribosomal subunit protein uL15 (RPL27A)	739.05	2.00 × 10^−4^	0.047	0.56	
	Myocardial zonula adherens protein (MYZAP)	1227.59	2.00 × 10^−4^	0.047	0.514	
	Palladin (PALLD)	1687.3	3.00 × 10^−4^	0.048	−0.326	
	Supervillin (SVIL)	903.31	3.00 × 10^−4^	0.049	0.411	Septal thickness: r = 0.8452, *p* = 0.0062

Clinical correlations = Spearman r value; LVEDV = left ventricular end diastolic volume; LVEF = left ventricular ejection fraction; LVESV = left ventricular end systolic volume; NF = no heart failure; DCM = dilated cardiomyopathy; oHCM = obstructive hypertrophic cardiomyopathy; * = duplicate entry—top 10 up-regulated and unique to oHCM.

**Table 2:** Significant protein expression changes that are associated with clinical features of obstructive HCM.

Protein Name (Gene)	%CV	p adj DCM v oHCM	log2ratio DCM/oHCM	p adj ISCM v oHCM	log2ratio ISCM/oHCM	Clinical Correlations
Cytochrome c oxidase subunit 7A-related protein, mitochondrial (COX7A2L)	5.6	0.001	0.091	0.004	−0.476	RVSP: r = −0.7381; *p* = 0.0458
Complement C1q subcomponent subunit A (C1QA)	6.1	0.044	−0.243	0.010	0.766	LV Mass: r = 0.8333; *p* = 0.0154
Complement C2 (C2)	3.1	0.023	0.444	0.007	0.838	LV Mass: r = 0.7857, *p* = 0.0279
Pyruvate dehydrogenase E1 component subunit alpha, somatic form, mitochondrial (PDAH1)	2.4	0.033	0.047	0.017	−0.280	LVEDV: r = 0.7667, *p* = 0.0214
Complement component C7 (C7)	6.1	0.004	−0.374	0.007	1.184	LVEDD: r = 0.7888, *p* = 0.0036
Collagen alpha-2(VI) chain (COL6A2)	2.6	0.046	−0.118	0.001	1.045	LVEF: r = 0.7224, *p* = 0.0098
Enoyl-CoA hydratase domain-containing protein 2, mitochondrial (ECHDC2)	6.1	0.037	−0.310	0.010	−0.415	BNP: r = 0–1.000, *p* = 0.0167
Zinc finger protein 397 (ZNF397)	20.7	0.041	0.256	0.013	−0.444	LVEDV: r = 0.8833, *p* = 0.0031
Complement factor D (CFD)	9.9	0.034	0.358	0.008	1.255	LV Mass: r = 0.7619; *p* = 0.0368 Septal thickness: r = 0.7197, *p* = 0.0338
Complement C1q subcomponent subunit C (C1QC)	6.0	0.026	−0.260	0.008	0.928	LVEDD: r = 0.6991, *p* = 0.0143 Post-amyl LVOT gradient: r = 0.8214, *p* = 0.0341
Complement component C9 (C9)	5.0	0.009	−0.129	0.008	0.998	LVEDV: r = −0.7381, *p* = 0.0458 RVSP: r = 0.8571, *p* = 0.0107
C4b-binding protein alpha chain (C4BPA)	4.5	0.031	0.000	0.005	0.991	LVEDD: r = 0.5988, *p* = 0.0431
Attractin (ATRN)	7.4	0.016	−0.009	0.015	0.433	LV Mass: r = 0.9048, *p* = 0.0046
Ceruloplasmin (CP)	6.1	0.005	0.259	0.020	0.854	
Alpha-1-antitrypsin (SERPINA1)	2.9	0.004	−0.426	0.005	0.932	
Alpha-1-antichymotrypsin (SERPINA3)	4.5	0.019	−1.031	0.046	0.877	
Complement C3 (C3)	2.8	0.038	0.287	0.036	0.615	
Plasma protease C1 inhibitor (SERPING1)	5.1	0.007	−0.295	0.008	0.791	
Inter-alpha-trypsin inhibitor heavy chain H4 (ITIH4)	1.4	0.012	0.295	0.021	0.641	
Homeodomain-interacting protein kinase 2 (HIPK2)	21.7	0.001	−0.009	0.000	−0.648	

Clinical correlations = Spearman r value; RVSP = right ventricular systolic pressure; LV Mass = left ventricular mass; LVEDV = left ventricular end diastolic volume; LVEDD = left ventricular end diastolic diameter; LVEF = left ventricular ejection fraction; BNP = brain natriuretic peptide; LVOT = left ventricular outflow tract obstruction; DCM = dilated cardiomyopathy; oHCM = obstructive hypertrophic cardiomyopathy; ISCM = ischaemic cardiomyopathy.

## Data Availability

The mass spectrometry proteomics data have been deposited to the ProteomeXchange Consortium via the PRIDE [34] partner repository with the dataset identifier PXD054266.

## Supplementary Materials

The following supporting information can be downloaded at https://www.mdpi.com/article/10.3390/biomedicines12081712/s1. Table S1: Significantly differentially expressed proteins between oHCM and non-heart-failure patients. Table S2: Significantly differentially expressed proteins between oHCM and both ISCM and DCM. Table S3: Top up- and down-regulated proteins. Table S4: MS experimental run order.
