# Supplementary material for: Proteomic Characterisation of Heart Failure Reveals a Unique Molecular Phenotype for Hypertrophic Cardiomyopathy
Source: Biomedicines. 2024 Aug 1;12(8):1712. doi: 10.3390/biomedicines12081712 (PMC11351942; doi:10.3390/biomedicines12081712)
Supplement: Supplementary file 1 [file biomedicines-12-01712-s001.zip › Table S3_top up and down oHCM proteins.pdf]

Table S3. Selection of Significantly up and down-regulated Proteins in obstructive HCM compared to NF controls.

	Protein Name (Gene ID)	Median (NF, DCM, ISCM, oHCM)	p	p.adj	log2ratio	Clinical correlation
**Unique oHCM + validated**	Apolipoprotein A-IV (APOA4)*	1067.90	6E-07	0.004	1.299	
	N(G),N(G)-dimethylarginine dimethylaminohydrolase 1 (DDAH1)*	132.40	4E-05	0.027	1.116	**LVEDV:** r=-0.9048, *p=0.0046* **LVEF:** r=-0.7197, *p=0.0344* **LVESV:** r=0.7167, *p=0.0369*
	Fructose-bisphosphate aldolase A (ALDOA)	36406.44	1E-04	0.045	-0.235	**LVEDV:** r=0.8333, *p=0.0154*
	Ubiquitin domain-containing protein UBFD1 (UBFD1)	182.30	1E-04	0.045	-0.487	
	Serine/threonine-protein kinase PAK 2 (PAK2)	197.35	2E-04	0.047	-0.289	
	Beta-enolase (ENO3)	12210.88	2E-04	0.047	-0.419	
	Large ribosomal subunit protein uL15 (RPL27A)	739.05	2E-04	0.047	0.560	
	Myocardial zonula adherens protein (MYZAP)	1227.59	2E-04	0.047	0.514	
	Palladin (PALLD)	1687.30	3E-04	0.048	-0.326	
	Supervillin (SVIL)	903.31	3E-04	0.049	0.411	**Septal thickness:** r=0.8452, *p=0.0062*
**Top 10 most up-regulated**	Periostin (POSTN)	4833.69	3E-03	0.121	3.015	**LVEDV:** r=-0.8333, *p=0.0154*

	Protein Name (Gene ID)	Median (NF, DCM, ISCM, oHCM)	p	p.adj	log2ratio	Clinical correlation
	Fibromodulin (FMOD)	188.44	1E-02	0.213	2.176	**LVEDV:** r=-0.9048, *p=.00046* **Septal thickness:** r=0.7133, *p=0.0371*
	Hyaluronan and proteoglycan link protein 1 (HAPLN1)	164.50	2E-03	0.115	1.925	**LVEDV:** r=-0.9524, *p=0.0011*
	Microfibril-associated glycoprotein 4 (MFAP4)	370.69	9E-04	0.077	1.615	**LVEDV:** r=-0.8571, *p=0.0107* **septal thickness:** r=0.6862, *p=0.0470*
	Adipocyte enhancer-binding protein 1 (AEBP1)	490.94	1E-02	0.209	1.425	**LVEDV:** r=-1.000, *p<0.0001* **LVEF:** r=-0.7197, *p=0.0344*
	Cardiac phospholamban (PLN)	2827.75	9E-04	0.077	1.316	**LVEF:**r=0.5941, *p=0.0448*
	Apolipoprotein A-IV (APOA4)*	1067.90	6E-07	0.004	1.299	
	Latent-transforming growth factor beta-binding protein 2 (LTBP2)	975.59	4E-03	0.141	1.239	**LVEDV:** r=-0.9048, *p=0.0046* **LV Mass**: r=0.7381, *p=0.0458*
	Collagen alpha-1(XIV) chain (COL14A1)	3080.15	2E-02	0.274	1.137	
	N(G),N(G)-dimethylarginine dimethylaminohydrolase 1 (DDAH1)*	132.40	4E-05	0.027	1.116	**LVEDV:** r=-0.9048, *p=0.0046* **LVEF:** r=-0.7197, *p=0.0344* **LVESV**: r=0.7167, *p=0.0369*
**Top 10 most down regulated**	Myosin-6 (MYH6)	977.40	7E-03	0.176	-2.900	**LVEDV:** r=0.8095, *p=0.0218*
	Serum amyloid A-1 protein (SAA1)	64.05	4E-03	0.126	-2.819	**RVSP:** r=0.7857, *p=0.0279*
	[Pyruvate dehydrogenase (acetyl-transferring)] kinase isozyme 4, mitochondrial (PDK4)	400.97	1E-02	0.230	-2.632	

	Protein Name (Gene ID)	Median (NF, DCM, ISCM, oHCM)	p	p.adj	log2ratio	Clinical correlation
	Dehydrogenase/reductase SDR family member 7C (DHRS7C)	70.18	7E-03	0.176	-2.582	
	Phospholipase A2, membrane associated (PLA2G2A)	41.22	2E-03	0.115	-1.867	
	Scavenger receptor cysteine-rich type 1 protein M130 (CD163)	155.02	5E-04	0.058	-1.658	**Post-Amyl LVOT gradient**: r=0.8214, *p=0.0341*
	V-set and immunoglobulin domain-containing protein 4 (VSIG4)	13.32	7E-03	0.176	-1.615	**valsalva LVOT gradient:** r=0.8571, *p=0.0238* **septal thickness:** r=-0.7280, *p=0.0313*
	Sodium-coupled neutral amino acid symporter 2 (SLC38A2)	54.71	6E-03	0.158	-1.573	**LVEF:** r=0.7866, *p=0.0157* LVESV: r=-0.7333, *p=0.0311*
	Glycerol-3-phosphate dehydrogenase [NAD(+)], cytoplasmic (GPD1)	318.22	3E-03	0.116	-1.471	**RVSP:** r=-0.7619, *p=0.0368*
	Homeodomain-only protein (HOPX)	40.05	5E-02	0.378	-1.470	

Clinical correlations = spearman r value. RVSP, right ventricular systolic pressure; LV Mass, left ventricular mass; LVEDV, left ventricular end diastolic volume; LVEDD, left ventricular end diastolic diameter; LVEF, left ventricular ejection fraction; LVOT = left ventricular outflow tract obstruction; LVESV = left ventricular end systolic volume; NF = no heart failure; DCM = dilated cardiomyopathy; oHCM = obstructive hypertrophic cardiomyopathy; ISCM = ischemic cardiomyopathy; * = duplicate entry – top 10 up-regulated and unique to oHCM
